# Correction: Temporal phenotyping of neutrophils in post-cardiac arrest syndrome and extracorporeal membrane oxygenation-assisted resuscitation: A pilot study

**DOI:** 10.1371/journal.pone.0345138

**Published:** 2026-03-13

**Authors:** The PLOS One Staff

## Notice of Republication

This article was republished on March 1, 2026 to correct an error in the title that was introduced during the production process. The publisher apologizes for the error. Please download this article again to view the correct version. The originally published, uncorrected article and the republished, corrected articles are provided here for reference.

## Supporting information

S1 File Originally published, uncorrected article. (PDF)

S2 File Republished, corrected article. (PDF)
