# Supplementary material for: Temporal phenotyping of neutrophils in post-cardiac arrest syndrome and extracorporeal membrane oxygenation-assisted resuscitation: A pilot study
Source: PLoS One. 2025 Jul 31;20(7):e0329069. doi: 10.1371/journal.pone.0329069 (PMC12312883; doi:10.1371/journal.pone.0329069)
Supplement: S1 Table — (DOCX) [file pone.0329069.s001.docx]

**S1 Table. CyTOF Staining Panel.**

| **Marker** | **Clone** | **Isotope Label** |
| --- | --- | --- |
| CD45 | HI30 | ^89^Y |
| CD172ab | SE5A5 | ^111^Cd |
| CD8 | RPA T8 | ^112^Cd |
| CD20 | 2H7 | ^113^Cd |
| CD4 | RPA T4 | ^114^Cd |
| CD3 | IM7 | ^115^In |
| CD56 | NCAM16.2 | ^116^Cd |
| CD88 | S5/1 | ^141^Pr |
| CD11b | M1/70 | ^142^Nd |
| CD182 (CXCR2) | 5E8/CXCR2 | ^143^Nd |
| CD64 | 10.1 | ^144^Nd |
| CD16 | 3G8 | ^145^Nd |
| CD14 | M5E2 | ^146^Nd |
| CD13 | WM15 | ^147^Sm |
| TLR4 | 610015 | ^148^Nd |
| CD18 (a) | MEM-148 | ^149^Sm |
| CD11b (a) | CBRM1/5 | ^150^Nd |
| CD123 | 6H6 | ^151^Eu |
| CD170 | 1A5 | ^152^Sm |
| CXCR1 | 8F1 | ^153^Eu |
| CD15 | MC-480 | ^154^Sm |
| CD18 | TS1/18 | ^155^Gd |
| CD95-Fas | DX2 | ^156^Gd |
| BLT1 | 203/14F11 | ^158^Gd |
| CD11c | Bu15 | ^159^Tb |
| FPR1 | 350418 | ^160^Gd |
| CD162-PSGL1 | CHO131 | ^161^Dy |
| CD33 | WM53 | ^162^Dy |
| CD85j-ILT2 | GHI/75 | ^163^Dy |
| CD10 | HI10A | ^164^Dy |
| CD177 | MEM-166 | ^165^Ho |
| FPR3 | 374822 | ^166^Er |
| CD273 (PD-L2) | 24F.10C12 | ^167^Er |
| CD31 | WM59 | ^168^Er |
| CX3CR1 | REA385 | ^169^Tm |
| CD274-PD-L1 | 29E.2A3 | ^170^Er |
| CD66b | G10F5 | ^171^Yb |
| FPR2 | 304405 | ^172^Yb |
| C3AR | hC3aRZ8 | ^173^Yb |
| HLA-DR | L243 | ^174^Yb |
| CD54 | HA54 | ^175^Lu |
| CD302 | 771910 | ^176^Yb |
