# Supplementary material for: Temporal phenotyping of neutrophils in post-cardiac arrest syndrome and extracorporeal membrane oxygenation-assisted resuscitation: A pilot study
Source: PLoS One. 2025 Jul 31;20(7):e0329069. doi: 10.1371/journal.pone.0329069 (PMC12312883; doi:10.1371/journal.pone.0329069)
Supplement: S2 Table — (DOCX) [file pone.0329069.s002.docx]

**S2 Table. Antibiotics Administered to the Study Population.**

|  | **Day 0** | **Day 1** | **Day 3** | **Day 7** |
| --- | --- | --- | --- | --- |
| **ECMO 1** |  | Ceftriaxone 2 g/day | Ceftriaxone 2 g/day | Ceftriaxone 1 g/day |
| **ECMO 2** |  |  | Ceftriaxone 1 g/day |  |
| **ECMO 3** |  | Ampicillin 3 g twice a day | Ampicillin 3 g twice a day | Ampicillin 3 g twice a day |
| **Non-ECMO 1** |  |  |  | Amoxicillin 250 mg three times a day |
| **Non-ECMO 2** |  | Ceftriaxone 2 g/day | Cefazolin 1 g/day |  |
| **Non-ECMO 3** |  | Ceftriaxone 2 g/day | Ceftriaxone 2 g/day | Ceftriaxone 2 g/day |
| **Non-ECMO 4** |  |  |  |  |
| **Non-ECMO 5** |  | Ceftriaxone 2 g/day | Ceftriaxone 2 g/day |  |

ECMO, extracorporeal membrane oxygenation
