# Supplementary figures and images for: Temporal phenotyping of neutrophils in post-cardiac arrest syndrome and extracorporeal membrane oxygenation-assisted resuscitation: A pilot study
Source: PLoS One. 2025 Jul 31;20(7):e0329069. doi: 10.1371/journal.pone.0329069 (PMC12312883; doi:10.1371/journal.pone.0329069)

## Slide 1
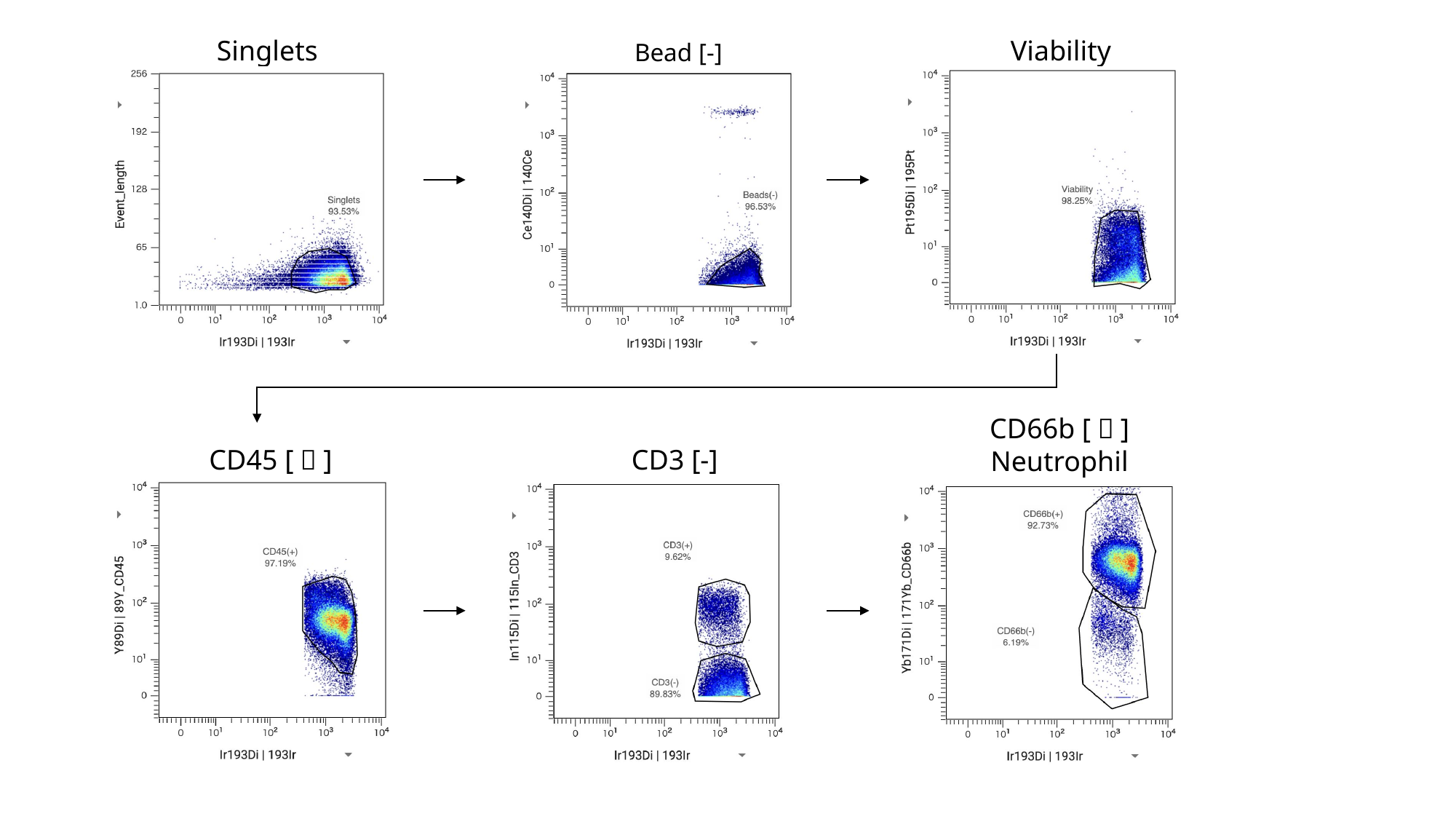

Singlets
Viability
Bead [-]
CD66b [＋]
Neutrophils
CD45 [＋]
CD3 [-]

Supplement: S1 Fig — (PPTX) [file pone.0329069.s003.pptx]
