# Supplementary material for: Correction: Temporal phenotyping of neutrophils in post-cardiac arrest syndrome and extracorporeal membrane oxygenation-assisted resuscitation: A pilot study
Source: PLoS One. 2026 Mar 13;21(3):e0345138. doi: 10.1371/journal.pone.0345138 (PMC12987479; doi:10.1371/journal.pone.0345138)
Supplement: S2 File — (PDF) [file pone.0345138.s002.pdf]

RESEARCH ARTICLE

Temporal phenotyping of neutrophils in post-cardiac arrest syndrome and extracorporeal membrane oxygenation-assisted resuscitation: A pilot study

Yuki Chiba[1], Asumi Mizugaki[1], Takumi Tsuchida[1], Katsuhide Kayano[2], Kazuma Yamakawa[2], Takeshi Wada[1]*

1 Division of Acute and Critical Care Medicine, Department of Anesthesiology and Critical Care Medicine, Faculty of Medicine, Hokkaido University, Sapporo, Japan, **2** Department of Emergency and Critical Care Medicine, Osaka Medical and Pharmaceutical University, Takatsuki, Osaka, Japan

* twada1@med.hokudai.ac.jp

Abstract

The role of extracorporeal membrane oxygenation in the immune response during cardiac arrest, as well as the role of the innate immune system—particularly neutrophils—in the pathophysiology of post-cardiac arrest syndrome, remains underexplored. This study aimed to comprehensively analyze the immune response in the pathology of post-cardiac arrest syndrome. This study included eight patients who experienced cardiogenic cardiopulmonary arrest and were treated for at least 1 week. Blood samples were collected immediately after the return of spontaneous circulation (day 0), as well as on days 1, 3, and 7. These patients formed the post-cardiac arrest syndrome group, and blood samples from five healthy volunteers served as controls. Neutrophil function over time was analyzed using CyTOF® mass cytometry. Furthermore, patients in the post-cardiac arrest syndrome group were divided into extracorporeal membrane oxygenation and non-extracorporeal membrane oxygenation groups according to whether they received extracorporeal membrane oxygenation during resuscitation. Cytokine levels were compared between the two groups using LUMINEX®. Three patients with post-cardiac arrest syndrome underwent extracorporeal membrane oxygenation. Compared with the control group, the post-cardiac arrest syndrome group showed fewer CD177-negative neutrophils and fewer strongly leukotriene B4 receptor 1-positive neutrophils. The extracorporeal membrane oxygenation group had more CD177-negative neutrophils and fewer CD16-seropositive neutrophils than the non-extracorporeal membrane oxygenation group. Differences in serum cytokine levels between the extracorporeal membrane oxygenation and non-extracorporeal membrane oxygenation groups were noted, with certain cytokines, including interleukin-6 and interleukin-8, decreasing over time only in the extracorporeal membrane oxygenation group. As the first in-depth immunological

Check for updates

OPEN ACCESS

Citation: Chiba Y, Mizugaki A, Tsuchida T, Kayano K, Yamakawa K, Wada T (2025) Temporal phenotyping of neutrophils in post-cardiac arrest syndrome and extracorporeal membrane oxygenation-assisted resuscitation: A pilot study. PLoS One 20(7): e0329069. https://doi.org/10.1371/journal.pone.0329069

Editor: Kamal Sharma, UN Mehta Institute of Cardiology and Research Center, INDIA

Received: November 17, 2024

Accepted: July 10, 2025

Published: July 31, 2025

Copyright: © 2025 Chiba et al. This is an open access article distributed under the terms of the Creative Commons Attribution License, which permits unrestricted use, distribution, and reproduction in any medium, provided the original author and source are credited.

Data availability statement: All relevant data are within the manuscript and its Supporting Information files.

Funding: TW JSPS KAKENHI (Grants-in-Aid [C] 20K09260, 21K09008, and 22K09109) and the Marumo Emergency Medicine Research

Promotion Fund (2020). https://kaken.nii.ac.jp/ja/grant/KAKENHI-PROJECT-20K09260/ https://kaken.nii.ac.jp/ja/grant/KAKENHI-PROJECT-21K09008/ https://kaken.nii.ac.jp/ja/grant/KAKENHI-PROJECT-22K09109/ https://www.jaam.jp/info/2019/info-20191219.html Did the sponsors or funders play any role in the study design, data collection and analysis, decision to publish, or preparation of the manuscript? ⇒No.

Competing interests: The authors declare no conflicts of interest related to the submitted work. TW received research funding from the Takeda Science Foundation, and personal fees for lectures from Asahi Kasei Pharma America Inc. and Asahi Kasei Pharma Japan Inc., all unrelated to the submitted work. This does not alter our adherence to PLOS ONE policies on sharing data and materials.

investigation of post-cardiac arrest syndrome, including neutrophil phenotyping, this study may inform clinical practices related to patient management and treatment strategies following cardiac arrest.

Introduction

Patients generally have a poor prognosis following cardiac arrest. The initial response during rescue and post-resuscitation treatment can greatly impact patients' subsequent quality of life. Various reactions occur throughout the body following cardiac arrest and the return of spontaneous circulation after a certain period, which are referred to as post-cardiac arrest syndrome (PCAS) [1]. In PCAS, damage-associated molecular patterns are synthesized, leading to the production of pro-inflammatory cytokines by binding to pattern-recognition receptors expressed on immune and endothelial cells [2]. Damage-associated molecular patterns induce platelet activation, which leads to neutrophil activation and the subsequent release of neutrophil extracellular traps (NETs) [2]. NETs mainly consist of histones, which promote the further release of NETs [3,4]. The synergistic interactions between NETs and histones lead to dysregulated inflammatory coagulofibrinolytic responses, resulting in multiple organ dysfunction syndrome [2,5]. Therefore, understanding the involvement of the innate immune system in PCAS—especially neutrophils, which play a central role in innate immunity—may be helpful for post-resuscitation treatment.

In recent years, the use of extracorporeal membrane oxygenation (ECMO) for patients with cardiac arrest who do not respond to general cardiopulmonary resuscitation has gained attention [6–8]. ECMO triggers the activation of the complement and contact systems upon exposure to foreign materials, leading to increased white blood cell activity and a systemic inflammatory response. However, the specific role of ECMO in the immune response during cardiac arrest is not fully understood [9].

CyTOF® is a powerful tool for high-dimensional and high-throughput single-cell assays. Recently, CyTOF® has attracted attention as a tool for elucidating the immune response in hematopoietic stem cell transplantation [10,11], cancer immunotherapy [12,13], and coronavirus disease 2019 [14,15]. Compared with conventional flow cytometry, CyTOF® uses rare earth metal isotope-labeled antibodies instead of fluorescent-tagged antibodies, which improves the discrimination between markers and increases the variety of cell identification markers [16]. In addition, LUMINEX-®100/200TM (hereinafter referred to as LUMINEX®) is a multi-parameter profiling technology that can simultaneously detect up to 80 cytokines in a sample volume of only 25 μL. This is achieved by reacting beads, colored with a fluorescent dye that specifically binds to the target protein, with the target antigen and measuring the reaction using flowmetry.

This study aimed to comprehensively analyze the immune response in the pathology of PCAS using CyTOF® and LUMINEX®, which are novel immune analysis tools.

Materials and methods

Study population

This study included patients with PCAS who visited Hokkaido University Hospital between September 2020 and October 2021. The study excluded patients aged <18 years, those who experienced non-cardiogenic cardiac arrest, and those without complete blood sample collection by the study date. Written informed consent for collecting patient data and blood samples was obtained from the family members or next of kin. Blood samples were collected at five time-points: immediately (day 0), 24 hours (day 1), 72 hours (day 3), and 7 days (day 7) after the return of spontaneous circulation. Data regarding clinical and biological parameters, such as demographic characteristics, the sequential organ failure assessment score, acute physiology and chronic health evaluation II score, and items related to the condition at the onset of PCAS, were collected from medical records and emergency service reports. For the control group, five healthy medical staff members of our hospital who provided informed consent were recruited. This study was conducted in accordance with the Declaration of Helsinki and approved by the Institutional Review Board of Hokkaido University School of Medicine (approval number: 018–0243).

Sample preparation

Two milliliters of ethylenediaminetetraacetic acid-infused blood samples were collected from eligible patients and consenting hospital staff members. The cells in the blood samples were conditioned in culture medium (C5: RPMI 1640, 5% heat-inactivated fetal calf serum, 1 mM glutamine, 10 mM HEPES, 2 mM non-essential amino acids, penicillin/streptomycin/fungizone, and 2.5×10^{-5} M 2 mercaptoethanol). C5 medium was purchased from Life Technologies Corporation (Carlsbad, CA, USA). Blood samples were washed and centrifuged repeatedly in C5 after red blood cell removal using our custom-formulated ammonium chloride-based red blood cell lysis buffer. Pellets containing cells accumulated at the bottom of the centrifuge tubes were collected by adding 0.5 ml cell freezing medium (CryoStor®; Biolife Solutions, Inc., Bothell, WA, USA) and then stored in tubes. The tubes containing cells were placed in a Mr. Frosty® container that had been previously cooled at 4°C. Tubes containing the cell-freezing medium were first cooled slowly in a refrigerator at 4°C to avoid rapid freezing. Twenty minutes of cooling was followed by overnight (12–24 hours) storage in a freezer at -80°C. Tubes in the Mr. Frosty container were immediately stored in a liquid nitrogen tank at -196°C until used for mass cytometry.

Mass cytometry

The CyTOF staining panel used in this study is shown in S1 Table. All CyTOF® staining procedures were performed at room temperature. Cisplatin viability staining reagent (Cell-IDTMCisplatin-198Pt; Fluidigm Sciences, South San Francisco, CA, USA) was added to the cells for 5 minutes and the cells were washed using centrifugation. Human TruStain FcX (BioLegend, San Diego, CA, USA) was added to the cells, and the mixture was incubated for 5 minutes. Subsequently, a CyTOF® antibody staining cocktail was added to the cells for 30 minutes. After staining, the cells were washed once with CyTOF® staining buffer (calcium/magnesium-free phosphate-buffered saline, 0.2% bovine serum albumin, 0.05% sodium azide). Next, the cells were incubated with a palladium-based barcode reagent for 30 minutes. The barcode reagent was washed off, and the specimens were pooled into a single tube. The cells were washed with an iridium intercalator solution (Max-Par Intercalator-Ir 500 mM; Fluidigm Sciences) for 20 minutes. The cells were then incubated with MilliQ-filtered distilled water (EMD Millipore, Billerica, MA, USA) at a concentration of 1×10^6 cells/mL, containing EQ calibration beads (EQ Four Element Calibration Beads; Fluidigm Sciences).

Utilizing the Normalizer and Single Cell Debarker software developed at the Nolan Lab (Stanford, Palo Alto, CA, USA), pooled single samples were analyzed using Helios™, a CyTOF®System (Fluidigm Sciences). Additionally, normalization and deconvolution were performed. Data from CyTOF® were subjected to multidimensional data analysis using OMIQ®

(Omiq, Inc., Santa Clara, CA, USA), which used optimized t-distributed stochastic neighbor embedding for the dimensionality compression algorithm and FlowSOM's auto-optimization parameters for the clustering and visualization algorithms. The number of meta-clusters (MCs) was set to the value that best separated the data based on the MC results from the elbow method. The results of optimized t-distributed stochastic neighbor embedding for each group were concatenated and compared.

Cytokine levels in the plasma

The obtained blood samples were centrifuged at 700 × g for 20 minutes at 25°C. The supernatants were collected and stored in a freezer at -20°C. The samples underwent multiplex analysis using the LUMINEX® system and the MILLIPLEX® MAP kit (Merck Millipore Corporation, Darmstadt, Germany).

Statistical analysis

Statistical analyses were conducted using JMP 16 Pro software (SAS Institute Inc., Cary, NC, USA). Data from patients with PCAS with and without ECMO were compared using the nonparametric Wilcoxon signed-rank test. Statistical significance was set at $P < 0.05$.

Results

Patient characteristics

This study enrolled eight patients with PCAS and five healthy controls. The patient group had no specific history of immunological or other diseases and immunosuppressive drug use. The demographic characteristics of patients included in this study are shown in Table 1. Patients with PCAS were divided into two groups: the ECMO group, consisting of three patients resuscitated with ECMO, and the non-ECMO group, consisting of five patients resuscitated without ECMO. The baseline characteristics were generally similar between the two groups; however, the low-flow time in the ECMO group was significantly longer than that in the non-ECMO group. Furthermore, the sequential organ failure assessment score in the ECMO group was significantly higher than that in the non-ECMO group. Two patients underwent decannulation and removal of ECMO the day after admission, and one patient did so 2 days after admission. The antibiotics administered to the study patients are shown in S2 Table.

Age, body mass index, CPR duration (Low-flow time and No-flow time), SOFA score, APACHE II score, lactate and white blood cell counts are presented as the median (range). Other items are shown as the number and percentage of participants.

Neutrophils contour plots and MCs

The gating strategy for neutrophils is shown in S1 Fig. Cells gated with cluster of differentiation (CD)-66b were identified as neutrophils and compared with the control and PCAS groups using a contour plot (Fig 1A). Visual differences between the control and PCAS groups were confirmed in zones A–D. Compared with the control group, the PCAS group had more cells in zone A and fewer cells in zone B. In the PCAS group, zone C showed a daily decrease, and zone D was fuller than in the control group.

PCAS, post-cardiac arrest syndrome; MC, meta cluster; CD, cluster of differentiation; BLT-1, leukotriene B4 receptor1; opt-SNE, optimized parameters for T-distributed stochastic neighbor embedding

We created a scatterplot to observe MCs using FlowSOM and identified zones A–D in the contour plot (Fig 1A). Zones A–D corresponded to MC-01, MC-17, MC-30, and MC-31, respectively (Fig 1B).

Next, we examined differences in the expression of markers between the major neutrophil population, MC-01, and individual cell populations in histograms to characterize individual cell populations (Fig 1C). CD177 expression differed

Table 1. **Baseline characteristics of patients with PCAS and those resuscitated with and without ECMO at admission to the emergency department.**

	PCAS (n = 8)	ECMO (n = 3)	Non-ECMO (n = 5)	P-value
Age (years)	59 (45–69)	61 (49–67)	57 (45–69)	1.00
Male sex, n (%)	6 (75)	3 (100)	3 (60)	0.13
Body mass index	25.1 (18.9–34.7)	24.5 (22.6–29.1)	25.6 (18.9–34.7)	0.77
Witnessed arrest, n (%)	5 (62.5)	3 (100)	2 (40.0)	0.05
Bystander CPR, n (%)	6 (75.0)	2 (66.7)	4 (80.0)	0.68
CPR duration				
Low-flow time (min)	26.5 (5–61)	54 (31–61)	19 (5–30)	0.04
No-flow time (min)	2.5 (0–5)	1 (0–5)	3 (1–5)	0.37
Cause of cardiac arrest, n (%)				
Acute coronary syndrome	5 (62.5)	1 (33.3)	4 (80.0)	0.18
Arrhythmia	3 (37.5)	2 (66.6)	1 (20.0)	0.18
Initial rhythm				
Sinus	1 (12.5)	0 (0)	1 (20.0)	0.31
VT	1 (12.5)	1 (33.3)	0 (0)	0.14
VF	5 (62.5)	1 (33.3)	4 (80.0)	0.18
Asystole	1 (12.5)	1 (33.3)	0 (0)	0.14
SOFA score after admission	8 (4–15)	14 (13–15)	6 (4–9)	0.04
APACHE II score	29.5 (21–38)	35 (31–38)	26 (21–33)	0.07
IABP use, n (%)	3 (37.5)	2 (66.6)	1 (20.0)	0.19
Lactate (mmol/L)	7.6 (4.5–11.2)	6.4 (6.1–11.2)	7.7 (4.5–10.4)	1.00
White blood cell count (1,000/μL)	13.1 (7.8–20.2)	13.7 (7.–20.2)	13.7 (7.9–19.7)	0.77
Nosocomial infection, n (%)	0 (0)	0 (0)	0 (0)	–
In-hospital mortality, n (%)	1 (12.5)	1 (33.3)	0 (0)	0.14

PCAS, postcardiac arrest syndrome; ECMO, extracorporeal membrane oxygenation; CPR, cardiopulmonary resuscitation; VT, ventricular tachycardia; VF, ventricular fibrillation; SOFA, sequential organ failure assessment; APACHE II, acute physiology and chronic health evaluation II; IABP, intra-aortic balloon pumping.

https://doi.org/10.1371/journal.pone.0329069.t001

between MC-01 and MC-17 cells, with MC-01 characterized as CD177 positive and MC-17 as CD177 negative. MC-30 cells were more strongly positive for the leukotriene B4 receptor 1 (BLT-1) than MC-01 cells. In contrast, MC-31 was weakly positive for CD11b.

For further analysis of the PCAS group, we divided the patients into ECMO (n = 3) and non-ECMO (n = 5) groups and again subsampled the cells that had been gated with CD66b so that the number of cells in each group was the same. Next, similar to Fig 1A, we created MCs using FlowSOM and identified different MCs using a histogram (Fig 2A and 2B). Zones E and F in Fig 2A increased in the ECMO group compared with those in the non-ECMO group, whereas zone F in the ECMO group decreased over time and was smaller than that in the non-ECMO group by day 7 (Fig 2A). Zone G decreased in the ECMO group compared with that in the non-ECMO group. Zone G of the ECMO group increased over time and was comparable with that of the non-ECMO group on day 7. The characteristics of zones E, F, and G were investigated using MCs and histograms by FlowSOM as in the comparison of the control and PCAS groups.

Zones E, F, and G were identified as MC-21, MC-26, and MC-31, respectively (Fig 2B). MC-21, MC-26, and MC-31 were compared with MC-03, the main neutrophil cluster.

MC-21 and MC-26 were characterized using histograms, and differences in CD177 and BLT-1 expression were observed (Fig 2C). No expression of CD177 was observed in either MC-21 or MC-26, while only MC-26 showed stronger expression of BLT-1 than MC-03. MC-31 cells were more strongly positive for CD16 compared with those of MC-03.

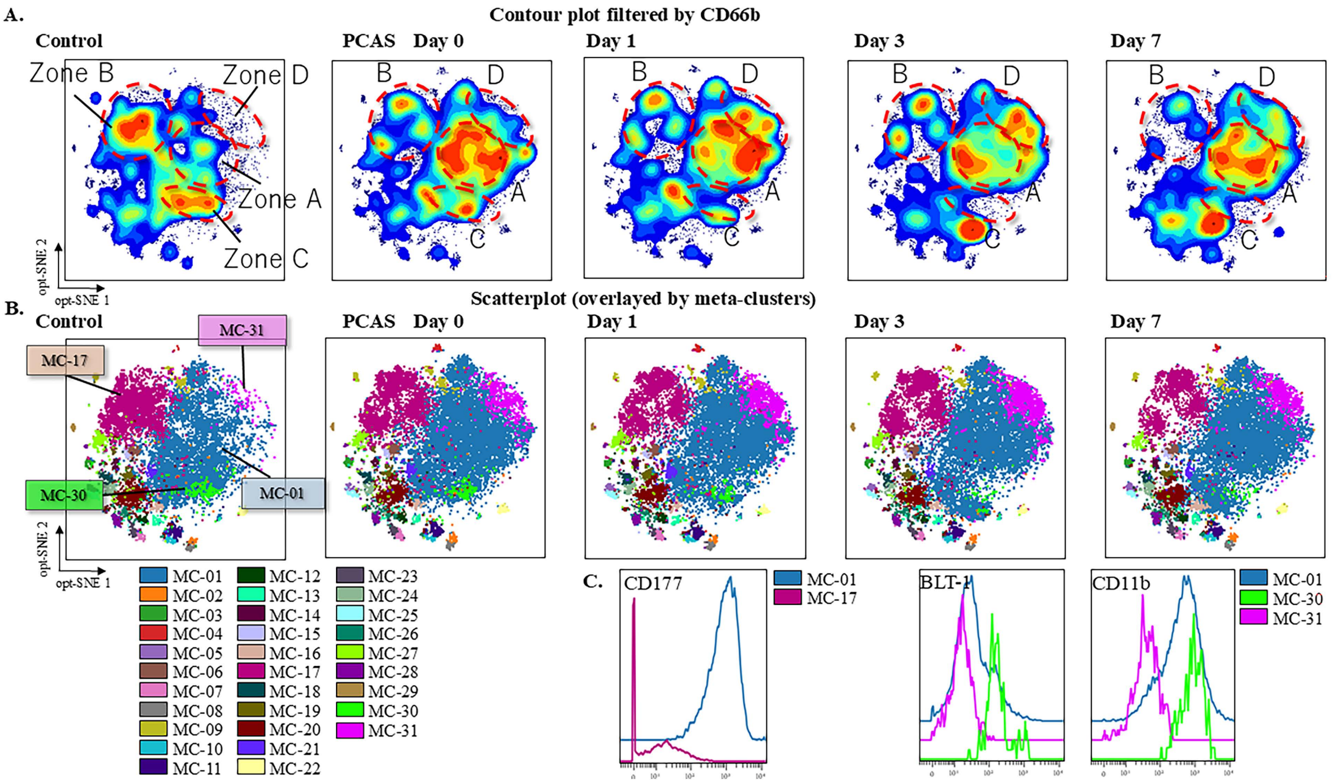

Fig 1. The Characteristics of Neutrophil Groups in the Control and PCAS Groups Are Illustrated by the Dimension Reduction Map. (A) The gating of CD66b is illustrated in the contour plot, with the files concatenated by groups. (B) Clustering by FlowSOM. (C) Histograms of CD177, BLT-1, and CD11b expression. In Fig 1B, we identified four distinct areas on the contour plot, labeled as Zones A–D. These zones correspond to specific MCs identified by FlowSOM analysis: Zone A corresponds to MC-01, Zone B to MC-17, Zone C to MC-30, and Zone D to MC-31. These zone labels were used to visually group the cell populations on the scatter plot, while the associated MC numbers were assigned by the FlowSOM algorithm based on marker expression profiles.

https://doi.org/10.1371/journal.pone.0329069.g001

Serum cytokine levels

LUMINEX® was used to observe differences in cytokine production with or without ECMO. We compared the same days between the ECMO and non-ECMO groups in the PCAS group.

Serum cytokine levels are shown in a heatmap (Fig 3A), which shows the relative relationship between the lowest value of 0 and the highest value of 100 for each item. The levels of granulocyte colony-stimulating factor, interleukin (IL)-10, IL-1 receptor antagonist, and interferon-γ inducible protein 10 in the ECMO group were elevated on day 0 and decreased by day 3 (Fig 3B). No changes were observed over time in the non-ECMO group. Other cytokines, such as IL-6 and IL-8, which are representative inflammatory cytokines, showed similar changes over time (Fig 3B).

Discussion

This study examined longitudinal changes in neutrophil function associated with PCAS pathology through a multidimensional analysis using CyTOF®. We observed that neutrophils had different expression levels of CD177, CD11b, and BLT-1 after cardiac arrest compared with those in the control group. In addition, resuscitation using ECMO may alter the expression of CD16, CD177, and BLT-1. Moreover, the induction of several cytokines by ECMO was confirmed through

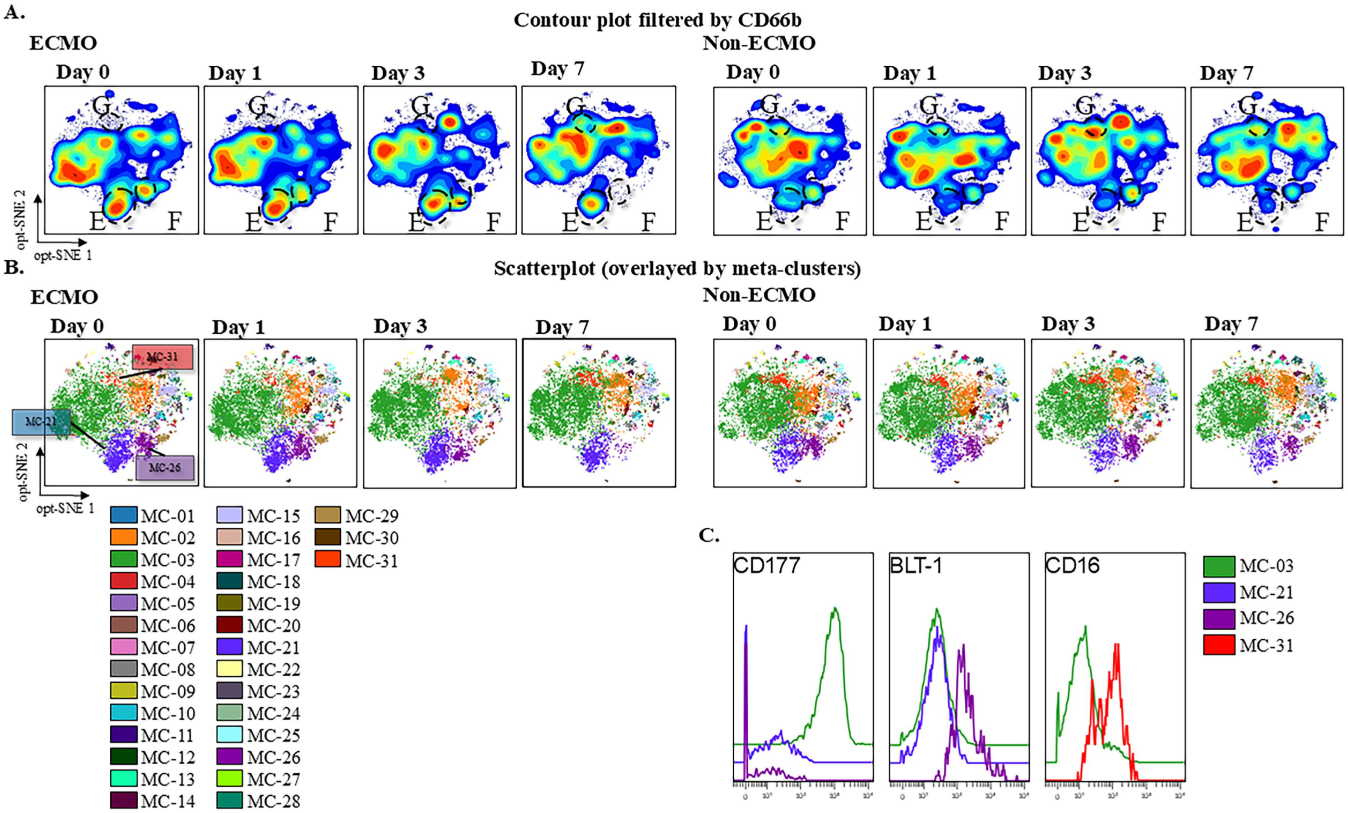

Fig 2. The Characteristics of Neutrophil Groups in the ECMO and Non-ECMO Groups Are Illustrated by the Dimension Reduction Map. (A) The gating of CD66b is illustrated in the contour plot, with the files concatenated by groups. (B) Clustering by FlowSOM. (C) Histograms of CD177, BLT-1, and CD16 expression. ECMO, extracorporeal membrane oxygenation; MC, meta cluster; CD, cluster of differentiation; BLT-1, leukotriene B4 receptor1; opt-SNE, optimized parameters for T-distributed stochastic neighbor embedding.

https://doi.org/10.1371/journal.pone.0329069.g002

LUMINEX®. To our knowledge, this is the first study to comprehensively evaluate the innate immune responses in patients with PCAS using novel immune analysis tools, including CyTOF® and LUMINEX®.

CD177-positive and -negative neutrophils in the PCAS group increased and decreased, respectively, compared with those in the control group (Fig 1C). CD177 remains an enigmatic expression marker of neutrophils. However, neutrophils carrying CD177 have been observed in various inflammatory diseases, and it is recognized as an inflammation-associated marker [17–20]. CD177-positive and -negative neutrophils are capable of migration; however, the binding of MEM166—an anti-CD177 antibody that mimics proteinase 3—to CD177 has been shown to arrest migration [21]. This suggests that CD177-positive neutrophils are less likely to migrate in PCAS due to the binding of CD177 with proteinase 3, a component of NETs, present in the blood in large quantities. In contrast, the migration of CD177-negative neutrophils occurs without any change in their migration ability. The decrease in CD177-negative neutrophils in the PCAS group could be due to an increase in CD177-positive neutrophils and a relative decrease in CD177-negative neutrophils as a result of systemic inflammation caused by PCAS or because CD177-negative neutrophils migrate to the tissues, resulting in a reduction in their levels in the blood.

Next, we observed that the number of MCs strongly expressing BLT-1 decreased in the PCAS group over time (Fig 1A). BLT-1 is a receptor for leukotriene B4, an important factor in migration; migration is initiated when leukotriene B4 binds to BLT-1 [22]. Neutrophils expressing BLT-1 were found to migrate into the infarcted area of the heart following myocardial

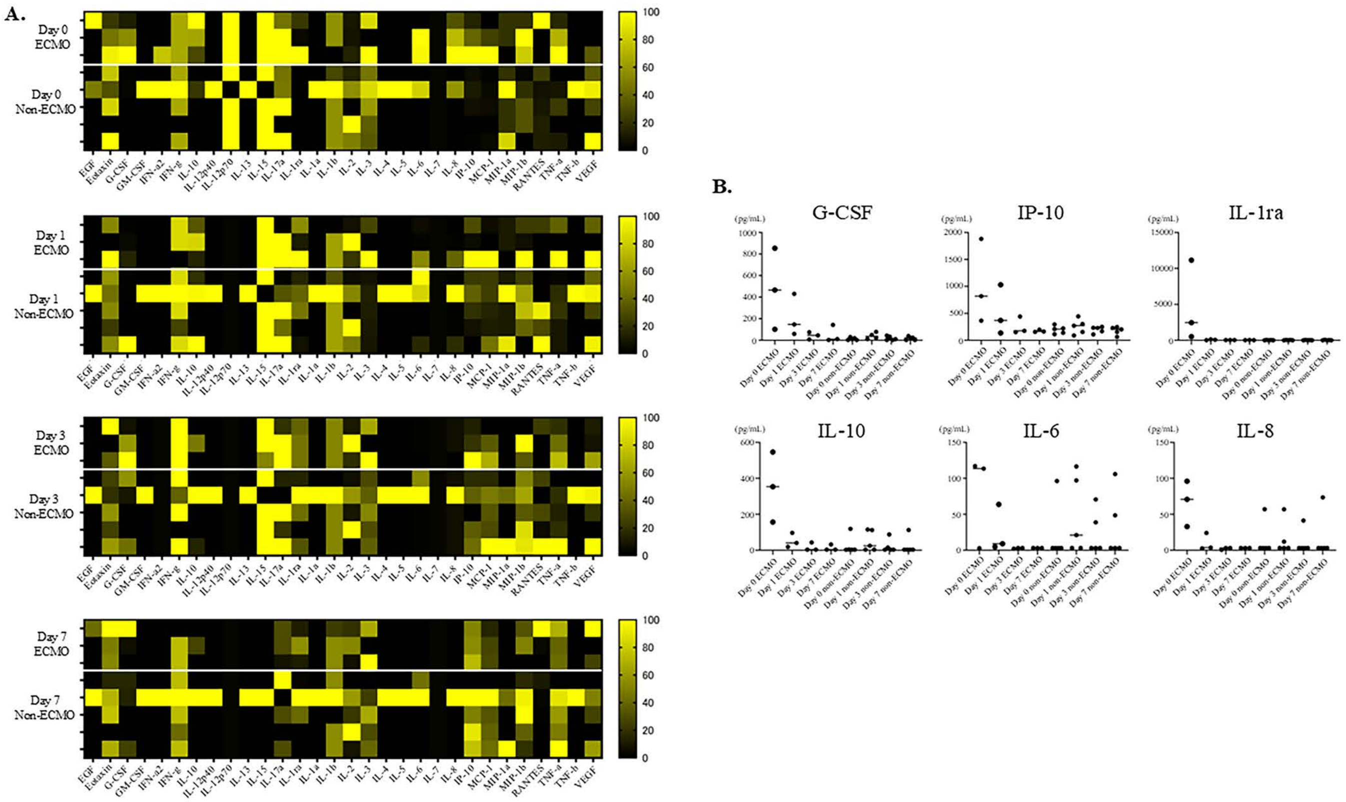

Fig 3. Serum Cytokine Levels in the ECMO and Non-ECMO Groups. (A) Heat map of serum cytokine levels. (B) Elevated cytokine levels in the ECMO group during the observation period. ECMO, extracorporeal membrane oxygenation; EGF, epidermal growth factor; G-CSF, granulocyte colony-stimulating factor; IFN, interferon; IL, interleukin; IP, interferon gamma-induced protein; MCP, monocyte chemotactic protein; MIP, macrophage inflammatory protein; RANTES, regulated on activation normal T cell expressed and secreted; TNF, tumor necrosis factor; VEGF, vascular endothelial growth factor.

https://doi.org/10.1371/journal.pone.0329069.g003

infarction [23]. The current study included only patients with cardiogenic cardiac arrest. Migration of neutrophils to the heart, similar to that in myocardial infarction, may have occurred in the PCAS group, potentially leading to a decrease in neutrophil levels.

Additionally, we compared differences in neutrophil functional alterations between the ECMO and non-ECMO groups. The levels of CD177-negative neutrophils showing strong BLT-1 positivity decreased over time in the ECMO group. This may be because CD177-negative neutrophils are less likely to undergo migration arrest, and neutrophils with strong BLT positivity have a higher migratory capacity. Therefore, CD177-negative neutrophils with strong BLT-1 positivity are more likely to undergo extravascular migration, leading to the observed results. The contact between blood and foreign material during ECMO may have resulted in an increased migratory capacity of neutrophils, characterized by the strong expression of BLT-1. In addition, neutrophils strongly expressing CD16 were observed to decrease in the non-ECMO group from days 0–3 but increased to the same level as in the non-ECMO group by day 7. Neutrophils that are strongly positive for CD16 are likely to be banded and segmented [24]. As patients on ECMO experience a decrease in peripheral blood leukocytes [25], the increase in adhesion factors associated with leukocyte activity may result in adhesion to the vascular endothelium or artificial lung surface, leading to a decrease in peripheral blood

leukocytes. However, as the present study did not examine the ECMO circuit, further assessment of neutrophil adherence and dynamics in the ECMO circuit is needed.

The mechanism of functional changes in the two types of aforementioned neutrophils (CD177-negative neutrophils with strong BLT-1 positivity and strongly CD16-positive neutrophils) in the ECMO group is not clear. However, a previous study reported that patients on ECMO have a higher prevalence of infections [26], suggesting that the decreased levels of two types of neutrophils (neutrophils with strong migratory ability and mature neutrophils) may be responsible for the increased susceptibility to infections. Several cytokines, including granulocyte colony-stimulating factor, interferon-γ inducible protein 10, IL-10, IL-6, and IL-8, were induced by ECMO, and their levels rapidly decreased to near-zero levels thereafter (Fig 3B). In contrast, IL-6 and IL-8, typical inflammatory cytokines, did not show a time-dependent decrease in blood levels in the non-ECMO group as observed in the ECMO group. These results may suggest that ECMO patients are unable to sustain appropriate inflammatory responses. Given that previous reports have shown a higher prevalence of infections in ECMO patients [26], this cytokine profile may contribute to an immunosuppressive or infection-prone state. However, this study had a small sample size and lacked data on the incidence of infections; therefore, we could not draw any conclusions regarding causality. These findings, although preliminary, may provide insight into a potential mechanism of ECMO-related immune dysfunction and underscore the need for further investigation with larger cohorts.

This study has some limitations. First, the study included a small number of patients. A total of 19 patients with PCAS were treated during the study period. However, to minimize variability in pathophysiology and prognosis, we only included patients with cardiogenic cardiac arrest. Cases of cardiac arrest due to non-cardiogenic causes—such as subarachnoid hemorrhage, asphyxia, and hanging—were excluded from the analysis. Second, the antibody panel used in this study was designed to phenotype immune cells, including not only neutrophils but also adaptive immune cells such as T and B cells; therefore, targeted evaluation of neutrophil function was not possible. Third, the ages and backgrounds of the participants in the control and PCAS groups were not matched, which may have resulted in large individual differences in the amounts of antibodies expressed in the cells. Fourth, the relationship between immune changes and organ failure revealed in this study was not further analyzed. In addition, because of the short duration of the study, the long-term prognosis, especially the neurological prognosis, could not be examined.

Conclusion

We observed several pathophysiological aspects of the innate immune response in patients with PCAS by phenotyping neutrophils over time using CyTOF® and conducting comprehensive cytokine measurements using LUMINEX®. Despite the small sample size, this is the first comprehensive immunological study, which may be positioned as a pilot study, to elucidate immunological aspects of PCAS pathologies.

Supporting information

S1 Table. CyTOF Staining Panel.
(DOCX)

S2 Table. Antibiotics Administered to the Study Population.
(DOCX)

S1 Fig. Gating Strategy.
(PPTX)

S1 Data. CyTOF fcs data1.
(ZIP)

S2 Data. CyTOF fcs data2.
(ZIP)

S3 Data. CyTOF fcs data3.
(ZIP)

S4 Data. CyTOF fcs data4.
(ZIP)

S5 Data. CyTOF fcs data5.
(ZIP)

Acknowledgments

We thank Shiho Kashiwabara for managing the laboratory equipment and Editage (https://www.editage.jp/) for English language editing.

Author contributions

Conceptualization: Takeshi Wada.

Data curation: Yuki Chiba, Asumi Mizugaki, Takeshi Wada.

Formal analysis: Yuki Chiba, Asumi Mizugaki, Katsuhide Kayano, Kazuma Yamakawa, Takeshi Wada.

Funding acquisition: Takeshi Wada.

Investigation: Asumi Mizugaki, Katsuhide Kayano, Kazuma Yamakawa, Takeshi Wada.

Methodology: Takumi Tsuchida, Katsuhide Kayano, Kazuma Yamakawa, Takeshi Wada.

Supervision: Takeshi Wada.

Validation: Asumi Mizugaki.

Writing – original draft: Yuki Chiba, Takeshi Wada.

Writing – review & editing: Asumi Mizugaki, Takumi Tsuchida, Katsuhide Kayano, Kazuma Yamakawa, Takeshi Wada.

References

1. Nolan JP, Neumar RW, Adrie C, Aibiki M, Berg RA, Böttiger BW, et al. Post-cardiac arrest syndrome: epidemiology, pathophysiology, treatment, and prognostication. A Scientific Statement from the International Liaison Committee on Resuscitation; the American Heart Association Emergency Cardiovascular Care Committee; the Council on Cardiovascular Surgery and Anesthesia; the Council on Cardiopulmonary, Perioperative, and Critical Care; the Council on Clinical Cardiology; the Council on Stroke. Resuscitation. 2008;79(3):350–79. https://doi.org/10.1016/j.resuscitation.2008.09.017 PMID: 18963350
2. Wada T. Coagulofibrinolytic Changes in Patients with Post-cardiac Arrest Syndrome. Front Med (Lausanne). 2017;4:156. https://doi.org/10.3389/fmed.2017.00156 PMID: 29034235
3. Thiam HR, Wong SL, Wagner DD, Waterman CM. Cellular Mechanisms of NETosis. Annu Rev Cell Dev Biol. 2020;36:191–218. https://doi.org/10.1146/annurev-cellbio-020520-111016 PMID: 32663035
4. Tan C, Aziz M, Wang P. The vitals of NETs. J Leukoc Biol. 2021;110(4):797–808. https://doi.org/10.1002/JLB.3RU0620-375R PMID: 33378572
5. Gando S, Wada T. Disseminated intravascular coagulation in cardiac arrest and resuscitation. J Thromb Haemost. 2019;17(8):1205–16. https://doi.org/10.1111/jth.14480 PMID: 31102491
6. Abrams D, MacLaren G, Lorusso R, Price S, Yannopoulos D, Vercaemst L, et al. Extracorporeal cardiopulmonary resuscitation in adults: evidence and implications. Intensive Care Med. 2022;48(1):1–15. https://doi.org/10.1007/s00134-021-06514-y PMID: 34505911
7. Wang C-H, Chou N-K, Becker LB, Lin J-W, Yu H-Y, Chi N-H, et al. Improved outcome of extracorporeal cardiopulmonary resuscitation for out-of-hospital cardiac arrest--a comparison with that for extracorporeal rescue for in-hospital cardiac arrest. Resuscitation. 2014;85(9):1219–24. https://doi.org/10.1016/j.resuscitation.2014.06.022 PMID: 24992872

8. Sakamoto T, Morimura N, Nagao K, Asai Y, Yokota H, Nara S. Extracorporeal cardiopulmonary resuscitation versus conventional cardiopulmonary resuscitation in adults with out-of-hospital cardiac arrest: a prospective observational study. Resuscitation. 2014;85:762–8.
9. Millar JE, Fanning JP, McDonald CI, McAuley DF, Fraser JF. The inflammatory response to extracorporeal membrane oxygenation (ECMO): a review of the pathophysiology. Crit Care. 2016;20(1):387. https://doi.org/10.1186/s13054-016-1570-4 PMID: 27890016
10. Matos TR, Hirakawa M, Alho AC, Neleman L, Graca L, Ritz J. Maturation and phenotypic heterogeneity of human CD4 regulatory T cells from birth to adulthood and after allogeneic stem cell transplantation. Front Immunol. 2020;11:570550.
11. McGuire HM, Rizzetto S, Withers BP, Clancy LE, Avdic S, Stern L, et al. Mass cytometry reveals immune signatures associated with cytomegalovirus (CMV) control in recipients of allogeneic haemopoietic stem cell transplant and CMV-specific T cells. Clin Transl Immunology. 2020;9(7):e1149. https://doi.org/10.1002/cti2.1149 PMID: 32642063
12. Lowther DE, Goods BA, Lucca LE, Lerner BA, Raddassi K, van Dijk D, et al. PD-1 marks dysfunctional regulatory T cells in malignant gliomas. JCI Insight. 2016;1(5):e85935. https://doi.org/10.1172/jci.insight.85935 PMID: 27182555
13. Krieg C, Nowicka M, Guglietta S, Schindler S, Hartmann FJ, Weber LM, et al. High-dimensional single-cell analysis predicts response to anti-PD-1 immunotherapy. Nat Med. 2018;24(2):144–53. https://doi.org/10.1038/nm.4466 PMID: 29309059
14. Mitsuyama Y, Yamakawa K, Kayano K, Maruyama M, Wada T, Fujimi S. Prolonged enhancement of cytotoxic T lymphocytes in the post-recovery state of severe COVID-19. J Intensive Care. 2021;9(1):76. https://doi.org/10.1186/s40560-021-00591-3 PMID: 34930468
15. Mitsuyama Y, Yamakawa K, Kayano K, Maruyama M, Umemura Y, Wada T. Residual persistence of cytotoxicity lymphocytes and regulatory T cells in patients with severe coronavirus disease 2019 over a 1-year recovery process. Acute Med Surg. 2022;9:e803.
16. Iyer A, Hamers AAJ, Pillai AB. CyTOF® for the Masses. Front Immunol. 2022;13:815828. https://doi.org/10.3389/fimmu.2022.815828 PMID: 35493491
17. Zhang R, Su L, Fu M, Wang Z, Tan L, Chen H, et al. CD177 cells produce neutrophil extracellular traps that promote biliary atresia. J Hepatol. 2022;77:1299–310.
18. Dahlstrand Rudin A, Amirbeagi F, Davidsson L, Khamzeh A, Thorbert Mros S, Thulin P, et al. The neutrophil subset defined by CD177 expression is preferentially recruited to gingival crevicular fluid in periodontitis. J Leukoc Biol. 2021;109(2):349–62. https://doi.org/10.1002/JLB.3A0520-081RR PMID: 32531826
19. Bonaventura A, Montecucco F, Dallegri F, Carbone F, Lüscher TF, Camici GG, et al. Novel findings in neutrophil biology and their impact on cardiovascular disease. Cardiovasc Res. 2019;115(8):1266–85. https://doi.org/10.1093/cvr/cvz084 PMID: 30918936
20. Zhou G, Yu L, Fang L, Yang W, Yu T, Miao Y, et al. CD177+ neutrophils as functionally activated neutrophils negatively regulate IBD. Gut. 2018;67(6):1052–63. https://doi.org/10.1136/gutjnl-2016-313535 PMID: 28468761
21. Bai M, Grieshaber-Bouyer R, Wang J, Schmider AB, Wilson ZS, Zeng L. CD177 modulates human neutrophil migration through activation-mediated integrin and chemoreceptor regulation. Blood. 2017;130:2092–100.
22. He R, Chen Y, Cai Q. The role of the LTB4-BLT1 axis in health and disease. Pharmacol Res. 2020;158:104857. https://doi.org/10.1016/j.phrs.2020.104857 PMID: 32439596
23. Horii Y, Nakaya M, Ohara H, Nishihara H, Watari K, Nagasaka A. Leukotriene B(4) receptor 1 exacerbates inflammation following myocardial infarction. FASEB J. 2020;34:8749–63.
24. McKenna E, Mhaonaigh AU, Wubben R, Dwivedi A, Hurley T, Kelly LA, et al. Neutrophils: Need for Standardized Nomenclature. Front Immunol. 2021;12:602963. https://doi.org/10.3389/fimmu.2021.602963 PMID: 33936029
25. Ki KK, Millar JE, Langguth D, Passmore MR, McDonald CI, Shekar K, et al. Current Understanding of Leukocyte Phenotypic and Functional Modulation During Extracorporeal Membrane Oxygenation: A Narrative Review. Front Immunol. 2021;11:600684. https://doi.org/10.3389/fimmu.2020.600684 PMID: 33488595
26. Tsangaris A, Alexy T, Kalra R, Kosmopoulos M, Elliott A, Bartos JA, et al. Overview of Veno-Arterial Extracorporeal Membrane Oxygenation (VA-ECMO) Support for the Management of Cardiogenic Shock. Front Cardiovasc Med. 2021;8:686558. https://doi.org/10.3389/fcvm.2021.686558 PMID: 34307500
